# Prevalence and risk factors of major depressive disorder in HIV/AIDS as seen in semi-urban Entebbe district, Uganda

**DOI:** 10.1186/1471-244X-11-205

**Published:** 2011-12-30

**Authors:** Eugene Kinyanda, Susan Hoskins, Juliet Nakku, Saira Nawaz, Vikram Patel

**Affiliations:** 1 Medical Research Council/Uganda Virus Research Institute, 50-59 Nakiwogo Street, Entebbe, Uganda & Senior Fellow of the European and Developing Countries Clinical Trials Partnership (EDCTP), Tygerberg 7505, Cape Town, South Africa; 2 Medical Research Council, Clinical Trials Unit, 125 Kingsway, London, UK; 3 Butabika National Psychiatric Referral Hospital, Off Old Port Bell Road, Kampala, Uganda; 4 Dartmouth Medical School, Dartmouth College, 1 Rope Ferry Road, Hanover, New Hampshire, USA; 5 London School of Hygiene & Tropical Medicine, Keppel Street, London, United Kingdom & Senior Wellcome Trust Fellow in Clinical Science, 215 Euston Road, London, UK

## Abstract

**Background:**

Not much is known about the risk factors of major depressive disorder (MDD) in HIV/AIDS in the African socio-cultural context. Therefore a study was undertaken to examine the prevalence and risk factors of MDD in HIV/AIDS in semi-urban Uganda.

**Methods:**

A cross-sectional study was undertaken among 618 respondents attending two HIV clinics in Uganda.

**Results:**

Prevalence of MDD was 8.1%. Factors associated with MDD at univariate analysis only were female gender, family history of mental illness, negative coping style, alcohol dependency disorder, food insecurity and stress; not associated with MDD were social support, neurocognitive impairment, CD4 counts and BMI. Factors independently associated with MDD were psychosocial impairment, adverse life events, post traumatic stress disorder, generalised anxiety disorder and life-time attempted suicide.

**Conclusion:**

Psychological and social factors were the main risk factors of MDD among ambulatory HIV positive persons with no evidence for the role of the neurotoxic effects of HIV. Treatment approaches for MDD in this patient group should be modeled on those used among non-HIV groups.

## Background

The increasing access to highly effective antiretroviral therapy (ART) for people living with HIV (PLWHA) even in low income countries including in sub-Saharan Africa has delayed HIV disease progression and prolonged survival bringing into sharp focus issues of quality of life including their mental wellbeing [1,2]. One of the major causes of psychiatric morbidity in HIV/AIDS is major depressive disorder (MDD), with studies from Africa reporting point prevalence rates of MDD of between 3% to 54% [3-8].

Most of these studies however were small, used non-locally validated screening scales to diagnose MDD and rarely examined risk factors.

Studies in the west have pointed to a multifactorial aetiology of MDD in HIV/AIDS including psychological, social and biological factors [9,10]. How much of these factors operate in the African socio-cultural environment is not fully known. The few studies that have been undertaken in sub-Saharan African point to the following risk factors for MDD in HIV/AIDS female gender, older age, unemployment, negative life events, childhood trauma, impaired function, poor social support, poor quality of life and low CD4 counts [5,6,11-13].

This paper examines the prevalence and risk factors of MDD in HIV/AIDS as seen in the African socio-cultural context of Uganda with a view to inform the development of mental health interventions for PLWHA on the African sub-continent.

## Methods

### Study design

This study was undertaken at two HIV clinics in the semi-urban district of Entebbe, Uganda. This study had two components a cross section study (from which this paper is derived) and a longitudinal follow-up component (still ongoing). In the cross sectional component of this study all consenting eligible HIV-infected patients attending two HIV clinics in the government health facilities of Entebbe District Hospital and Kigungu Health Centre III were continuously enrolled into this study. Eligibility criteria for inclusion in this study was that the individual must be registered with the study HIV clinics, aged 18 years and above, fluent in English or Luganda (the local language into which the study instruments had been translated) and not too physically and mentally sick (as examined by the attending clinician to undertake the interview). In this component of the study trained psychiatric nurses recruited from Uganda's National Psychiatric Referral hospital at Butabika conducted structured interviews to determine the prevalence and correlates of psychiatric disorders among the respondents.

### Data collection tools

The data collection tool for the cross sectional study component consisted of various structured and standardized modules which were translated into Luganda (the local language spoken in the study area), many of these modules were developed in the west have not been formally validated in the African context of Uganda. These tools were administered by trained psychiatric nurses, these included:

1) Socio-demographic factors sex, age, marital status, highest educational attainment, religion, and employment status.

2) Social factors i) '*food insecurity'*(assessed by the question, *'in the last month, did you or your family have enough food?'*); ii) *distance from the HIV clinic*; iii) *'duration of awareness of HIV status'*; iv) '*Negative life events score index'*, constructed from items of the adverse life events module of the European Parasuicide Interview Schedule that has previously been modified for the Ugandan situation by Kinyanda and colleagues (2005) [14,15]. For this study respondents were required to report whether they had experienced each of these events in the last 6 months. Items that were selected for inclusion in this study were those that were thought to be relevant to the HIV social situation in Uganda. The negative life events considered in this study looked at bereavement, severe illness and severe interpersonal conflict in the following significant social relationships parent, sibling, spouse/lover and child(ren). The individual related negative life events considered in this study examined for severe sickness, interpersonal conflict, feelings of isolation and abandonment, lack of the basic requirements of food, shelter and medicine, job loss, discrimination and worries about personal finances. A total score was generated to reflect the total number of life events reported, this scale had an α Cronbach of 0.82 in this study; v) '*Stress Score index'*, constructed by scoring each of the reported negative life events on a 3 point Likert scale where respondents were asked the question, *'how stressful did you find the event?' *with possible responses being: 0 = (not stressing/minimal stressing), 1 = (moderately stressing), 2 = (severely stressing). A total score was generated where high scores reflected more stress; vi) '*Social support index'*, constructed from items of the social support module of the European Parasuicide Interview Schedule [14]. This scale has four sub-scales each with 4 items that assess the following social support dimensions: 'need for social support', 'receive the required social support', 'felt needed for social support' and 'felt was providing the required social support'. Each of the items in these sub-scales is scored on a 3 point Likert scale where 1 = (not at all), 2 = (to some extent), and 3 = (yes, very much). Items of the sub-scale 'need for social support' were reverse scored so that higher scores on this indicated better social support just like the other three sub-scales. A total score was generated with higher scores reflecting better social support, the α Cronbach of this scale in this study was 0.84.

2) Psychological and clinical factors i) *Psychiatric disorder*was assessed using the M.I.N.I. neuropsychiatric interview (MINI Plus) which is a modular DSM IV based structured interview [16]. The psychiatric disorder modules used in this study were major depressive disorder, suicidality, alcohol use disorders, generalised anxiety disorder and post-traumatic stress disorder; ii) '*Negative coping style index'*was constructed from variables of the Mental Adjustment to Cancer Scale (MAC) whose items had been adapted to the local HIV situation [17]. Each of this scale's 17 questionnaire items is scored on a 4-item Likert scale 1 = (definitely does not apply to me), 2 = (does not apply to me), 3 = (applies to me), 4 = (definitely applies to me). To score all the questionnaire items so that they are all in the same direction i.e. higher scores reflecting more negative coping style required that questionnaires items (1,4,5,10,11,12, 13,15,16) that are cast positively such as item 1: *'I have been doing things that I believe will improve my health e.g. changed my diet.' *be reverse scored. A total score was generated so that higher scores reflected a more negative coping style, the α Cronbach of this scale in this study was 0.58; iii) '*past psychiatric history'*was assessed by the question, *'have you ever suffered from any nervous or psychiatric condition?*); iv) '*family history of psychiatric disorder'*; v) '*neurocognitive impairment*' assessed using the International HIV Dementia Scale [18]; vi) '*Psychosocial impairment index'*constructed from the three variables *'in the last month, on how many days were your normal activities disrupted through illness?' *(with responses scored as follows: none = score 0, one and above = score 1); *'how many times did you visit the health unit in the last month?' *(with responses scored as follows: none = score 0, one and above = score 1), *'for how many days were you admitted to hospital in the last month?' *(with responses scored as follows: none = score 0, one and above = score 1). A total score was generated so that higher scores reflected more impairment, this scale had an α Cronbach of 0.54; vii) *most recent CD4 count*; and viii) *body mass index*.

### Statistical analysis

**Figure 1:**
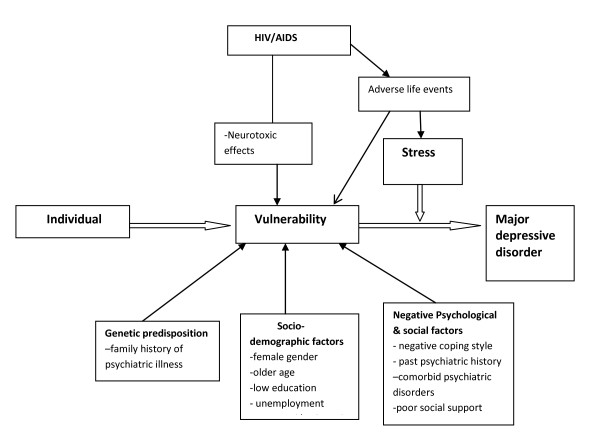
**Conceptual framework based on the Stress-Diathesis Model**.

A conceptual framework (Figure 1) based on the stress-vulnerability model for depression was specified a priori to guide the multivariate analyses [19].

Statistical analysis was undertaken using both SPSS (reliability tests) and STATA. Logistic regression models were used to assess univariate associations between the dependent variable (major depressive disorder) and independent variables, grouped in sets of demographic, social and psychological risk factors, with unadjusted and adjusted Odds ratio (adjusted for sex and age group) reported. After adjusting within each set of risk factors, those that were associated with MDD at a level of significance of 0.1 were entered into a multivariable model to determine their independent effect on MDD. Factors which remained associated with MDD at p < 0.05 were retained in the final model.

### Ethical Considerations

The study obtained ethical approval from the Uganda Virus Research Institute's Science and Ethics Committee, the Uganda National Council of Science and Technology and the London School of Hygiene Ethics Committee. Study participants were invited to consent after being provided with adequate information about the study. Respondents found to have significant psychiatric problems were referred to the psychiatric department at Entebbe district hospital for further assessment and management.

## Results

### Study population

From 6^th ^May 2010 to 10^th ^August 2010, 680 patients attending the HIV clinics at Entebbe hospital and Kigungu health centre were screened and given appointments to be interviewed for this study. Of these 618 (90.9%) were eventually enrolled into the study while 62 (4.9%) failed to turn up for their interview appointment even after being repeatedly contacted by telephone. Those who refused to participate in this study did not differ significantly from those who were enrolled into the study on gender and age. Of those who were enrolled into the study, Entebbe district hospital contributed 531 (85.9%) while the smaller Kigungu Health Centre III contributed 87 (14.1%). The population served by the two health centres was similar in terms of gender and age.

### Prevalence and socio-demographic risk factors of major depressive disorder

The prevalence of major depressive disorder in this study was 8.1% (95% CI, 5.9%- 10.2%).

Table 1 depicts the socio-demographic characteristics, with 449 (72.7%) females, and 455 (73.7%) patients in the 25-44 age band. Majority 553 (89.4%) of individuals had at least seven years of formal education but with only 53 (8.6%) having gone on to attain a tertiary education. 275 (48.5%) were married or currently living with someone, 75 (13.2%) widowed and 132 (23.3%) separated or divorced. Majority 262 (42.4%) were employed as small scale tradespersons, artisans or in the transport business (taxi drivers, taxi conductors and boda boda riders-motorcycle taxis).

**Table 1:** Socio-demographic correlates of Major Depressive Disorder

	Number in study (N,%)	Major depressive disorder (n,%)	Unadjusted OR (95% CI)	Adjusted OR^§^ (95% CI)
**Sex**			**P = 0.032**	**P = 0.077**
Male	169 (27.3)	7 (4.1)	1	1
Female	449 (72.7)	43 (9.6)	2.45 (1.08-5.56)	2.04 (0.88-4.75)
**Age (years)**			**P = 0.106**	**P = 0.018**
19-24	58 (9.4)	2 (3.4)	1	1
25-34	238 (38.6)	31 (13.0)	4.19 (0.97-18.06)	4.28 (0.99-18.46)
35-44	217 (35.1)	12 (5.5)	1.64 (0.36-7.54)	1.93 (0.42-8.96)
45+	103 (16.7)	5 (4.9)	1.43 (0.27-7.61)	1.69 (0.31-9.08)
**Marital status**			**P = 0.848**	**P = 0.936**
Currently married/cohabiting	275 (48.5)	23 (46.0)	1	1
Widowed	75 (13.2)	8 (16.0)	1.28 (0.55-2.97)	1.30 (0.52-3.24)
Separated/Divorced	132 (23.3)	11 (22.0)	0.99 (0.47-2.11)	0.91 (0.42-1.95)
Single	85 (15.0)	8 (16.0)	1.13 (0.49-2.61)	1.04 (0.44-2.46)
**Education level**			**P = 0.839**	**P = 0.545**
No education	65 (10.5)	7 (10.8)	1	1
Primary only	289 (46.8)	19 (6.6)	0.58 (0.23-1.45)	0.61 (0.24-1.55)
Secondary/above	264 (42.7)	24 (10.9)	0.83 (0.34-2.02)	0.98 (0.40-2.43)
**Religion**			**P = 0.494**	**P = 0.250**
Christians	535 (86.9)	45 (8.4)	1	1
Moslems	81 (13.1)	5 (6.2)	0.72 (0.30-1.87)	0.57 (0.21-1.49)
**Employment Status**			**P = 0.460**	**P = 0.171**
Farmer/Fisherman	97 (15.7)	10 (10.3)	1	1
Professional/clerical	43 (7.0)	2 (4.7)	0.42 (0.09-2.03)	0.37 (0.08-1.83)
Tradesperson/artisan/transport worker	262 (42.4)	21 (8.0)	0.76 (0.34-1.67)	0.53 (0.23-1.22)
Unemployed/house Wife	131 (21.2)	13 (9.9)	0.96 (0.40-2.29)	0.62 (0.24-1.59)
Others (including students)	84 (13.6)	4 (4.8)	0.43 (0.13-1.44)	0.35 (0.10-1.19)

**^§^**Adjusted for sex and age

On socio-demographic factors (Table 1), only female gender was associated with MDD in the unadjusted analysis. After adjusting for age, there was some evidence that female gender was associated with MDD (adjusted odds ratio (aOR) = 2.0, p = 0.08). Educational attainment, religion, marital status and occupation were not significantly associated with MDD.

### Social factors associated with major depressive disorder

Table 2 majority 358 (57.9%) lived 6 Km or more from the HIV clinic they were attending. Sixty nine (11.2%) respondents reported food insecurity for themselves and their families. Majority 465 (75.6%) had known their HIV status for 13 or more months, with most 399 (64.6%) on ART.

**Table 2:** Social correlates of Major Depressive Disorder

	Number in study (N,%)	Major depressive disorder (n,%)	Unadjusted OR (95% CI)	Adjusted OR^§^ (95% CI)
**Food Security**			**P = 0.004**	**P = 0.004**
Enough	549 (88.8)	38 (6.9)	1	1
Not enough	69 (11.2)	12 (17.4)	2.83 (1.40-5.73)	2.89 (1.40-5.98)
**Distance from HIV Clinic**			**P = 0.744**	**P = 0.673**
Less than 3 km	94 (15.2)	5 (5.3)	1	1
3 to 5 km	166 (26.9)	17 (10.2)	2.03 (0.72-5.69)	1.87 (0.66-5.30)
More than 5 km	358 (57.9)	28 (7.8)	1.51 (0.57-4.02)	1.51 (0.56-4.07)
**When knew HIV Status**			**P = 0.716**	**P = 0.912**
Up to 12 months ago	150 (24.4)	13 (8.7)	1	1
More than 12 months	465 (75.6)	36 (7.7)	1.13 (0.58-2.19)	1.04 (0.52-2.07)
**On ART**			**P = 0.482**	**P = 0.686**
Yes	399 (64.6)	30 (7.5)	0.81 (0.45-1.46)	0.88 (0.47-1.64)
No	219 (35.4)	20 (?)	1	1
**Social Support**			**P = 0.127**	**P = 0.236**
Low	188 (30.4)	20 (10.6)	1.59 (0.88-2.87)	1.44 (0.79-2.64)
High	430 (69.6)	30 (7.0)	1	1
**Negative life events**			**P < 0.001**	**P < 0.001**
1-5 events	350 (56.6)	8 (2.3)	1	1
6-10 events	191 (43.7)	29 (10.7)	4.72 (2.03-11.01)	4.29 (1.83-10.06)
11 +	77 (12.5)	23 (29.9)	18.21 (7.75-42.78)	16.67 (7.01-39.66)
**Stress Score index**			**P < 0.001**	**P < 0.001**
Low (score 0)	164 (26.5)	5 (3.0)	1	1
Medium (score 1-10)	323 (52.3)	18 (5.6)	1.88 (0.68-5.15)	1.74 (0.63-4.81)
High (score > 10)	131 (21.2)	27 (20.6)	8.26 (3.08-22.12)	7.18 (2.65-19.47)

**^§^**Adjusted for sex and age

After adjusting for age and sex, the social factors significantly associated with MDD were (Table 2): food insecurity (aOR 2.9, P = 0.004); increasing number of negative life events experienced in the last 6 months, for 6-10 events (aOR 4.3), 11+ events (aOR 16.7), P < 0.001 relative to the odds associated with reporting 1-5 events; and increasing stress scores, those with high stress scores (aOR 7.2), medium stress scores (aOR 1.7), P < 0.001 relative to the odds associated with low stress scores. Factors not significantly associated with MDD were: 'distance from HIV clinic', 'when got to know HIV status', 'being on ART', and social support. Furthermore, ORs for the association of stress scores, and of negative life events, and MDD did not change when adjusted for social support, indicating that social support did not moderate the effect of these factors.

Adjusting for negative life events, or increasing stress scores, greatly attenuated the association of female gender with MDD, indicating that the effect of gender was mediated through more proximate social factors. Women reported more negative life events than men (46% reporting 6+ events in past 6 months vs 36% of men), and higher stress scores (24% with high stress scores vs 13% of men).

### Psychological and clinical factors correlated with MDD

Table 3 depicts the psychological and clinical factors, 16(2.6%) had a past psychiatric history, while 120 (19.6%) had a family history of psychiatric illness. 396 (64.1%) had significant HIV associated neurocognitive impairment (a score of 10 or less on the International HIV Dementia Scale; Sacktor et al, 2005). The prevalence of psychiatric disorders/problems reported included post traumatic stress disorder 10 (1.6%), generalised anxiety disorder 5 (0.8%), alcohol dependency disorder 4 (0.7%) and life-time attempted suicide 24 (3.9%). On CD4 counts, 66 (12.5%) had < 100 cells/μL, 159 (30.2%) had 100-249 cells/μL while 220 (35.6%) had +350 cells/μL, the majority 387 (64.9%) had a normal BMI.

**Table 3:** Psychological and Clinical correlates of Major Depressive Disorder

	Number in study (N,%)	Major depressive disorder (n,%)	Unadjusted OR (95% CI)	Adjusted OR^§^ (95% CI)
				
**Negative Coping Style index**			**P = 0.002**	**P = 0.008**
Low	101 (16.3)	4 (4.0)	1	1
Medium	322 (52.1)	20 (6.2)	1.61 (0.54-4.81)	1.55 (0.51-4.68)
High	195 (31.6)	26 (13.3)	3.73 (1.26-11.01)	3.16 (1.06-9.43)
**Past Psychiatric history**			**P = 0.516**	**P = 0.570**
Present	16 (2.6)	2 (12.5)	1.65 (0.36-7.47)	1.56 (0.33-7.28)
**Family history of psychiatric illness**			**P = 0.003**	**P = 0.003**
Present	120 (19.6)	18 (15.0)	2.54 (1.37-4.71)	2.57 (1.37-4.83)
**Neurocognitive Impairment**			**P = 0.531**	**P = 0.535**
Present	396 (64.1)	30 (7.6)	0.83 (0.0.46-1.50)	0.83 (0.45-1.51)
**Psychosocial Impairment**			**P = 0.003**	**P = 0.003**
Present	318 (51.5)	36 (72.0)	2.61 (1.38-4.94)	2.65 (1.39-5.06)
**Life-time attempted suicide**			**P < 0.001**	**P < 0.001**
Present	24 (3.9)	13 (26.0)	17.79 (7.46-42.43)	17.51 (6.94-44.11)
**Post traumatic Stress disorder**			**P < 0.001**	**P < 0.001**
Present	10 (1.6)	8 (80.0)	53.91 (11.09-261.9)	43.76 (8.62-222.09)
**Generalized anxiety disorder**			**P = 0.001**	**P = 0.001**
Present	5 (0.81)	4 (80.0)	49.30 (5.40-450.3)	61.90 (5.90-649.0)
**Alcohol dependency disorder**			**P = 0.015**	**P = 0.003**
Present	4 (0.65)	2 (50.0)	11.79 (1.62-85.57)	24.35 (3.00-197.7)
**Most Recent CD4 count (cells/μL)**			**P = 0.413**	**P = 0.555**
< 100	66 (12.5)	4 (6.1)	1	1
100-249	159 (30.2)	11 (6.9)	1.15 (0.35-3.76)	1.14 (0.34-3.80)
250-349	81 (15.4)	7 (8.6)	1.47 (0.41-5.24)	1.39 (0.38-5.11)
350+	220 (35.6)	19 (8.6)	1.46 (0.48-4.47)	1.35 (0.43-4.24)
**BMI Index**			**P = 0.550**	**P = 0.288**
Underweight	55 (9.2)	4 (7.3)	1	1
Normal	387 (64.9)	36 (9.3)	1.33 (0.46-3.89)	1.07 (0.36-3.22)
Overweight	119 (20.0)	6 (5.0)	0.70 (0.19-2.60)	0.52 (0.13-1.97)
Obese	35 (5.9)	3 (8.6)	1.22 (0.26-5.80)	0.86 (0.17-4.30)

**^§^**Adjusted for sex and age

After adjusting for age and sex, factors significantly associated with MDD were: negative coping style index score (aOR 3.2 for high negative coping index score relative to low negative coping style index score, P = 0.008); a family history of psychiatric illness (aOR 2.6, P = 0.003); psychosocial impairment (aOR 2.7, P = 0.003); and the psychiatric disorders/problems of post traumatic stress disorder (aOR 43.8, P = < 0.001), generalised anxiety disorder (aOR 61.9, P = 0.001), alcohol dependency disorder (aOR 24.4, P = 0.003) and life-time attempted suicide (17.5, P < 0.001).

Factors not significantly associated with MDD were past history of psychiatric illness, HIV associated neurocognitive impairment, CD 4 counts and BMI.

### Factors associated with MDD at multivariate analysis

Table 4 in the final multivariable model, increasing number of negative life events, psychosocial impairment, life-time attempted suicide, post traumatic stress disorder and generalised anxiety disorder were independently associated with MDD at p < 0.05.

**Table 4:** Final Multivariate Model of risk factors for major depressive disorder in HIV/AIDS

	Odds ratio (95% CI)
**Negative life events**	**P = 0.004**
1-5 events	1
6-10 events	3.66 (1.51-8.86)
11 +	8.27 (3.18-21.46)
**Psychosocial Impairment**	**P = 0.014**
Present	2.41 (1.12-5.15)
**Life-time attempted suicide**	**P < 0.001**
Present	9.47 (3.36-26.73)
**Post traumatic Stress disorder**	**P = 0.003**
Present	14.07 (2.46-80.49)
**Generalized anxiety disorder**	**P = 0.028**
Present	20.01 (1.98-202.2)

## Discussion

The paper sought to investigate the prevalence and the psychological, social and biological risk factors of major depressive disorder (MDD) in HIV/AIDS in the African socio-cultural context. The principal finding of this study is that among ambulatory HIV/AIDS patients in the sub-Saharan African environment of Uganda, psychosocial impairment, increasing negative life events and the co-morbid psychiatric disorders/problems of post traumatic stress disorder, generalised anxiety disorder and life-time attempted suicide were the strongest independent determinants of MDD. Also associated with an increased risk for MDD in this study were the following psychological (family history of psychiatric disorder, negative coping style, alcohol dependency disorder), social (food insecurity, stress, poor social support) and socio-demographic (female gender) factors. In this study none of the investigated markers of direct HIV involvement including of the central nervous system (neurocognitive impairment, CD4 counts and BMI) were significantly associated with MDD. These results suggest while psychological and social factors were important risk factors for MDD among ambulatory HIV/AIDS persons in this socio-cultural environment, there was no evidence to support the role of the neurotoxic effects of HIV in MDD this sub-population of HIV/AIDS patients.

The prevalence of major depressive disorder obtained in this study was 8.1% a figure comparable to that of 9.6% reported by Chisanga et al (2011) in Zambia, 11.4% reported by Adewuya et al (2007) in Nigeria and 2.7% reported by Marwick and Kaaya (2010) in rural Tanzania [9,13,20]. All three studies above derived from sub-Saharan Africa used international diagnostic criteria to make a diagnosis of MDD just like we did in this study. However the picture on prevalence of MDD in HIV/AIDS is more complex than this, other studies undertaken both in South Africa and more recently in Uganda that used international diagnostic criteria reported rates of MDD as high as 40% [5,21]. This wide variability in rates of MDD has previously been noted by Judd and colleagues (1994) in a review of studies on MDD in HIV/AIDS [22]. Judd and colleagues (1994) attributed this wide variations in rates of MDD to a number of factors many of which are still relevant today, these included: methodological challenges in assessing MDD in somatically ill patients; differences in the composition of the study samples on 'at risk groups' (commercial sex workers, injection drug abusers, men who have sex with men, discordant couples, high risk occupational groups such as fisherfolk, and persons who acquired HIV perinatally) and on the different HIV clinical stages; whether the study population was predominantly in-patient or out-patient; and geographical differences [22]. Judd et al. (1994) summarised their observation by stating that it was not yet possible to come to a clear conclusion about the prevalence of MDD in HIV/AIDS, a summary which still seems to be pertinent to the sub-Saharan African setting today [22].

In this study female gender conferred a twofold increased odds for MDD relative to the male gender. A positive association between female gender and MDD in HIV/AIDS has previously been reported in Africa by both Kaharuza et al (2006) in Uganda and Orley et al (2004) in South Africa [6,12]. Some of the gender differences in MDD have been attributed to the more likelihood of females than males to become victims of traumatic experiences such as sexual, physical and emotional abuse both in childhood and in adulthood [23]. Indeed in this study females reported more negative life events than males and adjusting for the more proximal factors of negative life events and increasing stress scores greatly attenuated the association of female gender with MDD. Contrary to a common finding in research in this area [24], adjusting for social support in this study did not reduce the strong association between MDD and negative life events nor the strong association between MDD and stress scores indicating that social support did not moderate these effects in this study.

The significant association between MDD and food insecurity has previously been described in both low and high income settings [25,26]. The exact direction of causality between MDD in HIV/AIDS and food insecurity and the underlying mechanisms are not known and should be investigated. Possible explanatory mechanisms include food insecurity (in low income settings) leading to intense competition for scarce resources and in the process undermining social solidarity; other studies have pointed to a possible role for the associated micronutrient deficiency in the aetiology of MDD in HIV/AIDS [27,28].

In this study a negative coping style was associated with MDD. In HIV/AIDS, negative coping styles such as the use of avoidant strategies has been shown to be associated with MDD [29]. A positive family history of mental illness was shown to be associated with MDD in this study. Judd and colleagues (2005) in Australia observed a positive trend for the influence of a family history of psychiatric problems and MDD although this did not attain statistical significance [11]. The observation in this study of a significant association between family history of mental illness and MDD suggests a possible role for genetic predisposition to MDD in this sub-population of HIV/AIDS patients. Various genetic polymorphisms including those involving the serotonin transporter gene have been previously associated with increased vulnerability to MDD in the general population in the context of psychosocial stress [30]. There is however need to determine the specific mechanisms underlying genetic vulnerability to MDD among ambulatory HIV/AIDS patients in this environment. Psychosocial impairment was significantly associated with MDD in this study. In both Orley's studies in South Africa, impaired psychosocial function was significantly associated with MDD [4,11]. In this study just like in Orley's studies it was not possible to determine the direction of causality between impaired psychosocial function and depression due to the cross-sectional study design.

In this study comorbid psychiatric disorders/problems of PTSD, generalized anxiety disorder, alcohol dependency disorder and life-time attempted suicide were significantly associated with MDD. Given the cross sectional design of this study, it was not possible to determine the direction of causality between these comorbid psychiatric disorders and MDD in this study. However in a prospective study in the US, Atkinson and colleagues (2008) reported that lifetime multiple psychiatric comobidity was the most potent predictor of incident MDD episodes among HIV infected men [31].

Neurocognitive impairment in this study, though high, was not associated with MDD. A similar observation was made by Atkinson and colleagues (2009) in the U.S.A. and Judd and colleagues (2005) in Australia [11,31]. None of the markers of HIV disease progression (CD4 counts and BMI) was significantly associated with MDD in this study. Akena and colleagues (2010) in their study in Uganda observed no association between MDD and CD4 counts [32]. Ciesla and Roberts (2001) in a meta-analysis involving 10 studies concluded that HIV stage was not a factor in MDD [33].

The findings from this study whereby neurocognitive impairment and markers of HIV disease progression were not significantly associated with MDD suggest that there is a minimal role for the neurotoxic effects of the HIV virus in the aetiology of MDD in ambulatory HIV patients in this socio-cultural environment. Judd and colleagues (2005) in Australia among 'well' PLWHA made a similar observation [11]. However to confirm this observation will require the study of more specific and sensitive markers of HIV associated inflammation such as the pro-inflammatory proteins [10].

There is however evidence from studies both undertaken in Uganda and an earlier study undertaken in the US for a possible neurotoxic role of HIV in the aetiology of MDD [10,34,35]. Two study groups in Uganda (with one involving the author, EK) have reported evidence for HIV associated organic affective disorder syndromes among HIV positive persons with severe mental illness [34,35]. Earlier in the US, Lyketsos and colleagues (1996) in a longitudinal study reported a dramatic sustained rise in depressive symptoms18-24 months before the onset of clinical AIDS a phenomenon they attributed to the widespread dysregulation of the immune system around this period [10]. These results taken together suggest a possible neurotoxic role of HIV in the aetiology of MDD. There is however need for more research in this area to better understand this phenomenon.

## Conclusions

The results from this study similar to what Judd and colleagues (2005) have reported in Australia suggest that the main risk factors for MDD among ambulatory PLWHA are psychological and social factors with no evidence for the neurotoxic effects of the HIV virus [11]. Arising from these results we can recommend that among ambulatory PLWHA in this socio-cultural environment, treatment approaches of MDD in HIV/AIDS should be modeled on those used for treating MDD in non-HIV groups. There is however need for more studies to confirm these findings in other sub-Saharan African settings.

Limitations of this study include firstly, that the cross sectional nature of this study made it difficult to determine the direction of causality of the factors associated with MDD. Therefore, there is need for longitudinal studies to establish the exact causal direction between MDD and many of the investigated variables. Secondly, a number of the tools used to assess various psychosocial constructs have not been locally validated. These tools were however locally adapted through a forward and backward translation process and to minimise bias, only those tools with a minimum α Cronbach of 0.50 were used in the analysis for this paper.

## List of abbreviations

EK: Eugene Kinyanda; SH: Susan Hoskins; JN: Juliet Nakku; SN: Saira Nawaz; VP: Vikram Patel.

## Competing interests

The authors declare that they have no competing interests.

## Authors' contributions

Concept: EK, SH, VP; Data collection: EK, SH, JN; Data analysis: EK, SN; First draft: EK, SH, JN, VP; Final revision: EK, SH, JN, VP, SN; All authors read and approved the final manuscript.

## Pre-publication history

The pre-publication history for this paper can be accessed here:

http://www.biomedcentral.com/1471-244X/11/205/prepub
